# How do changes to the built environment influence walking behaviors? a longitudinal study within a university campus in Hong Kong

**DOI:** 10.1186/1476-072X-13-28

**Published:** 2014-07-28

**Authors:** Guibo Sun, Nicolas M Oreskovic, Hui Lin

**Affiliations:** 1 Institute of Space and Earth Information Science, The Chinese University of Hong Kong, Shatin, Hong Kong; 2 Center for Child & Adolescent Health Research and Policy, Massachusetts General Hospital, Boston, MA, USA; 3 Departments of Internal Medicine and Pediatrics, Massachusetts General Hospital, Boston, MA, USA; 4 Harvard Medical School, Boston, MA, USA; 5 Department of Geography and Resource Management, The Chinese University of Hong Kong, Shatin, Hong Kong

**Keywords:** Longitudinal study, Built environment, Walking diary, Geographic information system

## Abstract

**Background:**

Previous studies testing the association between the built environment and walking behavior have been largely cross-sectional and have yielded mixed results. This study reports on a natural experiment in which changes to the built environment were implemented at a university campus in Hong Kong. Longitudinal data on walking behaviors were collected using surveys, one before and one after changes to the built environment, to test the influence of changes to the built environment on walking behavior.

**Methods:**

Built environment data are from a university campus in Hong Kong, and include land use, campus bus services, pedestrian network, and population density data collected from campus maps, the university developmental office, and field surveys. Walking behavior data were collected at baseline in March 2012 (n = 198) and after changes to the built environment from the same cohort of subjects in December 2012 (n = 169) using a walking diary. Geographic information systems (GIS) was used to map walking routes and built environment variables, and compare each subject’s walking behaviors and built environment exposure before and after the changes to the built environment. Walking behavior outcomes were changes in: i) walking distance, ii) destination-oriented walking, and iii) walked altitude range. Multivariable linear regression models were used to test for associations between changes to the built environment and changes in walking behaviors.

**Results:**

Greater pedestrian network connectivity predicted longer walking distances and an increased likelihood of walking as a means of transportation. The increased use of recreational (vs. work) buildings, largely located at mid-range altitudes, as well as increased population density predicted greater walking distances.Having more bus services and a greater population density encouraged people to increase their walked altitude range.

**Conclusions:**

In this longitudinal study, changes to the built environment were associated with changes in walking behaviors. Use of GIS combined with walking diaries presents a practical method for mapping and measuring changes in the built environment and walking behaviors, respectively. Additional longitudinal studies can help clarify the relationships between the built environment and walking behaviors identified in this natural experiment.

## Background

Walking is an important form of daily physical activity with many known health benefits, including improvements to cardiovascular health, lung function, bone strength, and mental health, and a decreased risk of diabetes, obesity, cancer, and overall mortality. At least 150 minutes of moderate or vigorous physical activity per week is recommended in adults in order to promote and maintain health [1,2]. Efforts to address low levels of physical activity have more recently focused on community-level characteristics. The built environment, “comprised of urban design, land use patterns, and transportation system and encompassing patterns of human activity within the physical environment”, [3] has become a prominent characteristic of interest. By studying and shaping the built environment, urban planners and health practitioners aim to encourage walking behaviors as a way to promote public health [4,5].

Empirical studies quantifying the relationship between the built environment and walking behavior have been largely cross-sectional [6]. Potential confounding factors, including walking context, residential self-selection [7] and socio-economics, along with the cross-sectional nature of the studies, have resulted in mixed findings on the interplay between the built environment and walking behavior [8]. A few studies have used quasi-longitudinal data to examine the relationship between the built environment and active travel (walking) behavior [9,10]. However, recall bias after the respondent’s relocation remained a noted limitation. A natural experiment, where proposed upcoming changes to the built environment are known to occur, could provide an ideal venue for studying the effect of changes to the built environment on a study population, provided data were recorded from subjects both before and after the changes occurred. When substantial changes to the built environment occur over time, if well measured, it may be possible to identify the influence of the built environment on walking behavior or other health conditions, such as obesity [11].

There remains no consistent or agreed upon set of measures to assess changes to the built environment [12]. The so called “D” variables are classic objective measures of the built environment [6,13]. Density and diversity are land use indicators in conjunction with transport systems. In higher-density neighborhoods, land use is compact and destinations are closer, making walking more feasible and advantageous. Diversity indicates a mix of land use. Being equipped with more land use types that are within walking distance is considered to be favorable for walking. Design refers to the aesthetic or quality of the land use and the streetscape, including the presence and attractiveness of natural sights, recreational facilities, and architectural design. The D variables, however, may be necessary but not sufficient to explain people’s interactions with the built environment. People who live in the same neighborhood have different utilizations of the built environment [14]. This may be particularly true when changes occur to the built environment, people may adopt different walking and travel patterns to navigate their new surroundings. Individual-based measures of exposure to walking environments might provide a means to assess the role of destinations in the context of changes to the built environment, and help to investigate the association with changes to walking behaviors.

Accurately and consistently measuring walking behavior is also important. Self-reported measures have been variously quantified as estimates of the time spent in walking for transport and recreation, walked distances, and adherence to moderate-intensity physical activity recommendations on most of day of the week [15]. Self-reported assessments must be interpreted with caution, however, given the possibility of recall bias and other response errors [15,16]. More recently, global positioning system (GPS) has been proposed as a promising technological tool for objectively measuring individual behavior in terms of physical and transport-related activity [17]. Signal loss can be a limitation to using GPS in hilly and dense environments, however, especially in ultra-dense and hilly cities like Hong Kong. Travel diaries, including the Household Travel Survey, ask respondents to keep a log of detailed trips made during a particular time period, usually one or two days. The diaries usually include information on origin and destination, mode of travel, trip duration, and the primary activities at trip destinations, but information on walking data are frequently missed. Alternatively, data on walking trips obtained from a modified walking-oriented travel diary can provide an accurate individual-based measure of a subject’s interaction with the built environment.

We conducted a longitudinal study which collected built environment and walking behavior data within the context of a changing built environment. A natural experiment, whereby a university campus in Hong Kong experienced changes in the built environment, served as the study site. The changes to the built environment include changes to land use, campus bus services, pedestrian network, and population density. Baseline and follow-up surveys were collected from participants before and after changes to the built environment. An individual-based exposure measure of walking environment was designed and analyzed in GIS along with a walking diary.

## Methods

### Study area, population, and research design

The Chinese University of Hong Kong (CUHK) is chosen as a study location. Similar to the situation found in hilly topographical cities (e.g. Hong Kong), this university campus possesses a complex 3-dimensional topological layout. Express lifts, outdoor escalators, foot bridges and hilly stairs on the CUHK campus directly link three height levels (from 0 to 157 meters in height). The steeply contoured site has resulted in the dispersal of buildings and formed nine colleges, each constituting a collegial neighborhood with its own hostels, dining halls and other facilities. Ten campus bus lines are in service. According to Hong Kong Government policy, CUHK was to revert from a three-year to a four-year program during the 2012/13 academic year, resulting in a 30% increase in enrollment. To accommodate this policy, the existing land use and transportation schedule on campus are being adjusted, and pedestrian networks are being increased or repaired. The activities concerned in our study are limited to those taking place on campus. Two travel modes are considered: walking and bus transportation (campus bus service).

**Figure 1:**
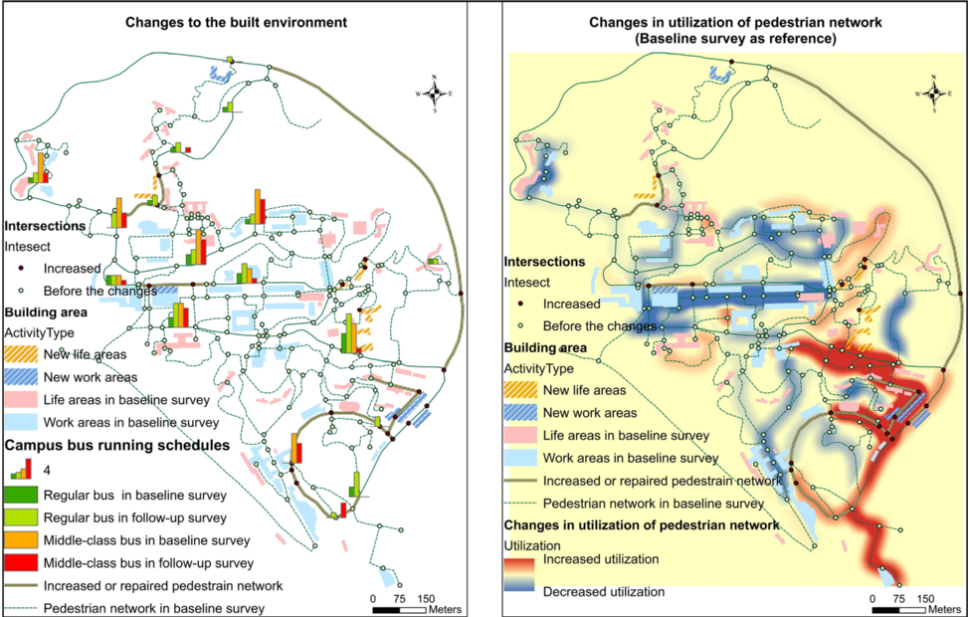
Changes to the built environment (left) and changes in utilization of pedestrian network (right).

Data on changes to the built environment were collected from the Campus Development Office of CUHK and were supplemented by field surveys. Four built environment change variables were created for analysis: change in land use, change in pedestrian network connectivity, change in bus schedules, and change in population density. Details of the changes to the built environment are listed in Table 1. A campus map after the built environment changes were made is shown in Figure 1 (Figure 1, left). The changes in the built environment make CUHK an ideal natural experiment in which to collect longitudinal pre-post built environment and walking behavior data on a cohort of subjects experiencing the changes.

**Table 1:** Changes to the built environment

	**Amount**
**Changes to land use**	
Increased number of buildings	14
Increased work area^a^	49216 m^2^
Increased life area^b^	59288 m^2^
**Changes to pedestrian network**	
Increased and repaired pedestrian network distance	2595 meters
Increased pedestrian network intersections	22
Increased (new/addition of) escalator^c^	1
**Changes to bus schedules**	
Increased bus stations	3
Changes to regular campus bus^d^ stops	25
Changes to middle-class campus bus^e^ stops	−18
**Changes in population density**	
Increased population	3456 persons (30% more)

^a^work area = includes buildings containing classrooms, laboratories and libraries.

^b^life area = includes buildings containing canteens, dormitories, public open space, and sport centers.

^c^Increased (new/addition of) escalator: before changes to the built environment, there were no escalators; a new outdoor escalator was installed for the purpose of facilitating engagement of the hilly topography at the Chinese University of Hong Kong.

^d^‘Regular bus’ are those available for all university staff, postgraduates and undergraduates.

^e^‘Middle-class bus’ are those available for undergraduates and commuters.

University students matriculated at the university during the time in which the built environment changes were scheduled to occur were eligible to participate. A convenience sample of students was recruited by mail and asked to complete two surveys, one before and one after the changes to the built environment. The CUHK Survey and Behavioral Research Ethics Committee approved the study. Written informed consent was obtained from all participants. We collected baseline built environment and survey data in March 2012 (n = 198, corresponded to 3% of eligible registered university undergraduate students (Mean = 18.7 years old, SD = 1.2)), 4 months prior to changes in the built environment. Changes to the built environment took place from August to September 2012, after which newly established buildings, pedestrian network and bus schedule were open, along with an increase in the first year student class size, which accounted for a 30% increase in the campus population density. Follow-up built environment and survey data were collected in December 2012 on the same cohort of subjects (n = 169, response rate = 85.36%), 3 months after changes to the built environment were completed.

### Walking diary: measurement of walking behavior

A walking-oriented travel dairy was developed for this study which collected information on each walking trip’s start and end time, walking route, social context (whether walking alone or with friends), and purpose (the activity at the destination).

A campus map with a detailed pedestrian network was distributed to each subject. It should be noted that the pedestrian network for walking is quite different than the street network for driving at the university campus; use of a traditional GIS street network database would be overly coarse and insufficient to adequately trace pedestrian paths on campus. Ideally, pedestrian networks should incorporate formal and informal paths, including sidewalks, laneways, pedestrian bridges, and park paths that are informal but frequently used for transportation. The missing pedestrian paths in the street network databases are likely to be the ones that are frequently used and which greatly increase the connectivity of dispersed destinations. Most studies on accessibility and connectivity use street networks only in their analyses, which may provide an incomplete description and prediction of walking behaviors [18], especially for a campus setting. For this study, in addition to use of traditional street networks, we developed a pedestrian network dataset by digitizing CUHK campus maps supplemented by field surveys. From these data, a pedestrian map was designed, with each road segment being numbered, to allow subjects to record detailed information on their use of the pedestrian network.

Daily walking trips were obtained from the walking diary and mapped in GIS (ArcGIS 10.2, ESRI, Redlands, California, USA) according to road segment numbers. Each subject could have multiple walking or bus transportation trips throughout their daily activities, thus providing repeated measures data, or, ‘one-to-many’ data as classified in ArcGIS. We conducted a Table Join by the ‘one-to-many’ relationship class in ArcGIS. Three walking behavior outcomes were created using this data: The walking distance, walking ratio (the number of a subject’s daily walking trips divided by the number of total (walking and vehicular) daily trips), and walked altitude range.

### Kernel density analysis: a qualitative analysis of changes in walking behavior

Statistical testing for changes in walking behavior typically reports mean difference values along with standard deviations. While such analytic methods that use aggregated data are powerful tools for identifying population-level changes, they lack the ability to capture individual-based interactions between a subject and his/her walking environment. Maps offer the capability to interpret the disaggregated spatial interactions via visualization.In the current study, we first extracted all of the walking trips in the baseline and follow-up surveys from the walking diaries. Next, the accumulated utilization of pedestrian network was calculated for the two survey waves by the frequency of use of each road segment in the pedestrian network. Kernel density estimates were then created using the Line Kernel density tool in ArcGIS with the cell size set at 50 meters. From the survey data collected before-and-after changes to the built environment, a map containing changes to utilization of every road segment in the pedestrian network was generated with the Raster Calculator tool in ArcGIS, by subtracting the follow-up from the baseline kernel density map (Figure 1, right). Geospatial and qualitative interpretations of changes in the walking behaviors are analyzed within the contexts of changes to land use, an increase in pedestrian connectivity, an adjustment of bus schedules and an increase in population density.

### Buffer and intersect analysis: individual-based measures of exposure to the built environment

We sought to determine how subjects interacted with their walking environment by using individual-based measurements of exposure to the built environment to test the interaction between subjects and their walking environment, in a disaggregated way.

After mapping the subjects’ walking diaries, we conducted a polyline Buffer analysis of each subject’s daily walking trips. It should be noted that different delimitations of contextual units may lead to different results on the effects of environmental factors on health behaviors or outcomes [19]. There is limited empirical evidence to identify the threshold within which people perceive the built environment as walking contexts or potential places of activity. In this study, we adopted Duncan’s suggested threshold while using a more tolerant buffer (e.g. If the buffer interacted with portion of a building area, we will count the whole building area as the potential places of activity area), in order to capture more detail about the exposure to changes in the built environment across our study area. Fifty meters buffers around street and path centerlines were used to capture subjects’ walking environments and potential places of activity [20]. The baseline and follow-up built environment exposures, before and after changes to the built environment, respectively, were mapped for each subject. We separated buildings by their land use function, into buildings primarily used for working (*work area*) or for living (*life area*), and also accounted for the building capacity. *Work area* buildings were classified as those containing classrooms, laboratories and libraries, while *life area* buildings included canteens, dormitories, public open space, and sport centers. Each subject’s built environment exposure was determined by overlaying a subject’s walking trip buffer areas onto the land use patterns. Changes to the pedestrian network, including increased and repaired paths, are represented as intersections. Exposure to bus stations was calculated separately for regular bus schedules which are available for all university staff, postgraduates and undergraduates, and for middle class bus schedules which are available for undergraduates and commuters. Given an underlying assumption of an evenly distributed population density throughout the campus, exposure to population density was calculated by multiplying the change in population ratio and the change in the pedestrian network used ratio, where the change in population ratio was from 100% to 130% and the change in pedestrian network use ratio is calculated by dividing the daily walking distance of a subject and total pedestrian network on campus.

### Statistical analysis

Statistical analyses were conducted in SPSS 20. Univariate analyses examined the distribution of the data and were used to calculate descriptive statistics. Data were inspected for outliers, with a g value set at 2.2 [21] leaving 146 subjects in the study sample for statistical analysis. Bivariate analyses using t-tests were used to test for changes in walking behaviors by comparing baseline and follow-up values for walking distance, walking ratio and walked altitude range.

Multivariable linear regression models adjusting for gender were run to test for the influence of changes in built environment exposures on changes in walking behaviors. We checked the final model for possible multicollinearity between the explanatory variables using Variance Inflation Factors (VIFs), and found that it was unlikely to be a problem (VIFs < 5). We conducted a heteroscedasticity test of the built environment characteristics in the final model using Koenker-test [22], concluding that it was also unlikely to be a problem (Chi-Square = 6.11, and p = 0.41). All built environment exposures variables were calculated as follow-up minus baseline exposures. Regression models included the changes to individual-based built environment exposures as the independent variables. Separate models were run for each dependent variable: change in walking distance, change in walking ratio, and change in walked altitude range, with significance set at p < 0.05.

## Results

### Sample characteristics

One-hundred ninety-eight subjects completed the baseline survey on March 2012 (Mean = 18.7 years old, SD = 1.2). After nine months, 169 of these subjects completed the second survey. Sample characteristics are reported in Table 2. The sample was evenly distributed by gender (56% female), and representative of the gender distribution at the Chinese University of Hong Kong.

**Table 2:** Sample characteristics

	**Baseline survey**		**Follow-up survey**	
	**n**	**%**	**n**	**%**
**Female**	109	55.05	95	56.21
**Male**	89	44.95	74	43.79
**Total**	198		169	

### A geospatial analysis of changes to walking behavior

**Figure 2:**
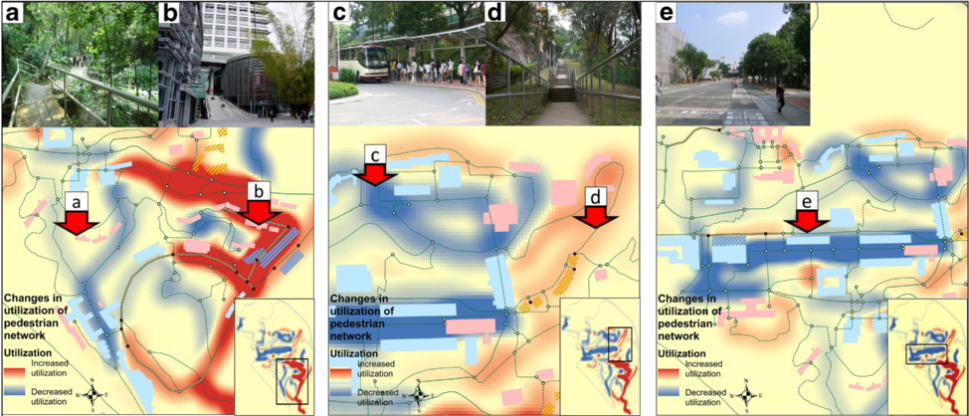
**Examples of changes to the built environment and to the utilizations of pedestrian network on campus. (a)** decreased utilization of a traditional pedestrian road segment, **(b)** new escalator and increased walking trips; **(c)** changes to a bus stop, from a start and end terminal to a pass-by terminal, **(d)** increased utilization of a traditional hilly stair pedestrian road segment; **(e)** decreased utilization of a traditional pedestrian road segment, around which is the traditional activity center.

Kernel density maps show the change of utilization of pedestrian network (Figure 1 right and Figure 2). Three kinds of walking behavior changes were identified: 1) a newly established escalator greatly encouraged walking trips from the lower-level to the lower-middle level of campus. The escalator and use of its connected pedestrian road segments increased, while utilization of the pre-existing alternative walking pedestrian road segments, despite having attractive walking environments, was decreased. The increased utilization of pedestrian networks contained most of the newly established or repaired pedestrian intersections; 2) Utilization of a traditional pedestrian hilly road containing stairs was increased. This increase may be related to a decrease in the scheduled frequency of a nearby campus bus which likewise occurred as part of the built environment changes on campus. Prior to the changes, a nearby bus station served as the start and terminus. After the changes, the bus station served only as a bus stop location. The wait time between scheduled buses also increased. 3) Utilization of a pedestrian road segment in the central part of campus was decreased. This may be related to a 14 building increase in the middle and lower campus levels, and suggests that that trips may be generated from type of activity. The center of activity on campus appears to have shifted after changes to the built environment were instituted; use of the old activity center decreased along with decreased use of the surrounding road segment.

### Individual-based measures of exposure to the built environment

**Figure 3:**
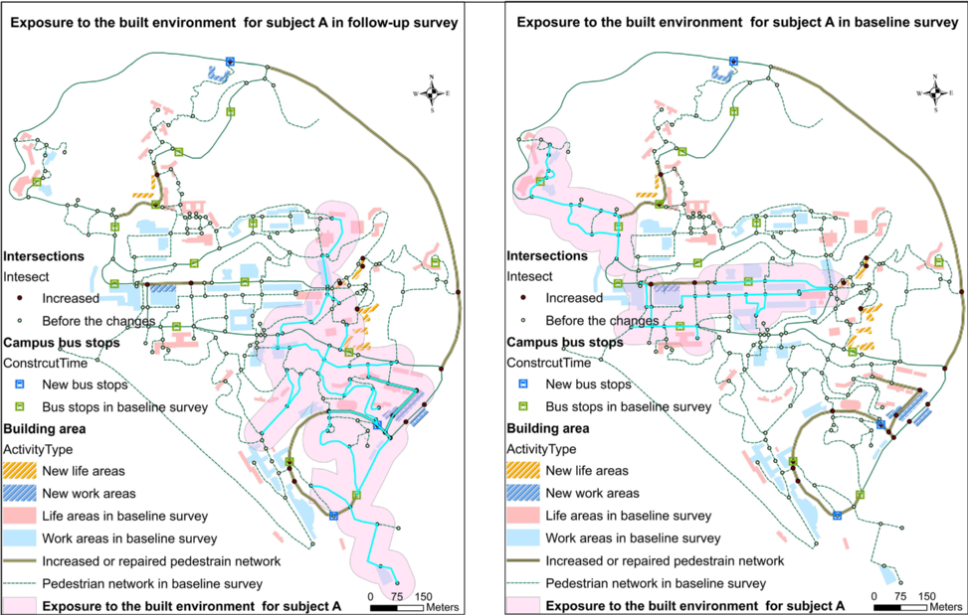
**Individual-based measure of exposure to the built environment.** An example of one subject’s change in exposure to the built environment using the baseline and follow-up survey (The lines highlighted in cyan are the daily walking behaviors for subject A; the polygon buffers highlighted in pink are the exposures to the built environment).

Individual-based daily walking trips for each subject were extracted from their walking diary and matched to the pedestrian network. Figure 3 shows one subject’s changes in exposure to the walking environment at baseline and follow-up after the changes to the built environment. Descriptive statistics reporting changes to the built environment and changes to walking behavior for all study subjects are listed in Table 3.

**Table 3:** Changes to the built environment and to walking behaviors (N = 146)

	**Baseline survey**		**Follow-up survey**		**Change**^**a**^		**p-value**^**b**^
	**Mean**	**SD**	**Mean**	**SD**	**Mean**	**SD**	
**Measures of walking behavior**							
Walking distance (meters)	3206.25	1176.51	3290.11	2019.95	83.86	2011.93	0.03
Walking ratio	0.89	0.55	0.9	0.92	0.01	0.11	0.02
Walked altitude range (meters)	100.42	96.5	99.2	64.19	−1.22	42.56	0.05
**Measures of the built environment**							
Exposure to work area	165487.94	59754.43	177728.62	63550.89	32072.23	77222.46	0.04
Exposure to life area	54951.15	25023.8	54786.02	24717.66	20847.84	32900.71	n.s.^c^
Exposure to pedestrian network intersection	54.42	29.73	62.75	32.91	7.9	38.56	0.01
Exposure to regular bus stops	5.82	5.17	16.93	10.57	10.64	10.87	<0.001
Exposure to middle-class bus stops	10.76	10.56	6.31	5.29	−3.64	9.48	<0.001
Exposure to population density	0.34	0.12	0.43	0.16	0.1	0.17	<0.001

^a^Each subject’s change in exposure is calculated using follow-up survey minus baseline survey data.

^b^*p* refers to the result of *t-test* comparing the mean in the baseline survey with that in the follow-up survey.

^c^n.s. = not significant; *p > .05*.

### Association between changes to the built environment and changes in walking behavior

The baseline survey is the reference category for each variable. The results of the multivariable linear regression models are listed in Table 4. An increase in pedestrian network connectivity, as measured by road intersections, positively predicted walking distance and walking ratio (p < 0.001 for both). An increased exposure to life area buildings, most of them located at the middle campus elevation level, and an increase in population density were associated with longer walks (p < 0.001 for both). At the lower campus level, an increase in the number of work area buildings was associated with a decrease in subjects’ altitude ranges (p < 0.001), while the increased bus service resulted in more people frequenting higher elevation levels (p = 0.01), and a change in middle-class bus service was inversely associated with subjects’ predicted movement across hilly terrain (p = 0.02). An increase in the population density was also found to positively predict a subject’s walking altitude range (p < 0.001). No gender differences were present.

**Table 4:** Multivariable linear regression models examining the influence of changes to the built environment on walking behavior (N = 146)

	**Changes to walking distance**			**Changes to walking ratio**			**Changes to altitude range**		
	**β**	**SE**	***p*-value**	**β**	**SE**	***p*-value**	**β**	**SE**	***p*-value**
**Change in exposure to building area**									
Change in exposure to work areas	−0.041	0.001	0.34	0.171	0 < 0.001	0.12	−0.385	0 < 0.001	<0.001**
Change in exposure to life areas	−0.147	0.003	<0.001**	−0.101	0 < 0.001	0.41	−0.152	0 < 0.001	0.18
**Change in exposure to pedestrian network**									
Change in exposure to pedestrian network intersection	0.895	2.177	<0.001**	0.408	0 < 0.001	<0.001**	−0.174	0.110	0.08
**Change in exposure to bus services**									
Change in exposure to regular bus stops	0.029	6.225	0.39	−0.037	0.001	0.67	0.208	0.314	0.01*
Change in exposure to middle-class bus stops	0.054	6.515	0.08	0.081	0.001	0.31	−0.175	0.328	0.02*
**Change in exposure to population density**									
Change in exposure to population density	0.187	724.549	<0.001**	0.115	0.107	0.48	0.996	36.529	<0.001**
Gender	0.008	112.174	0.76	−0.026	0.016	0.72	−0.024	5.655	0.72

SE = standard error.

*p < 0.05, **p < 0.001.

## Discussion

In this study, we present the results of longitudinal walking behaviors, before and after changes to the built environment occurring at a university campus in Hong Kong, and find that as exposure to the built environment changes, so do walking behaviors, including daily distances walked, the proportion of trips which are walked, and the altitude ranges walked by students.

Specifically, we found that an increase in pedestrian network connectivity, as measured by road intersections, was associated with a greater walking distance and walking ratio. Our findings are consistent with previous literatures showing that increased pedestrian network connectivity encourages walking for transportation [8,23-25]. For example, Berrigan, D. and his colleagues found that street connectivity accounted for a small but significant influence on active transportation [23], while the Twin Cities walking study also reported positive associations between street connectivity and walking for transportation [24,25]. Few studies, however, have been conducted in hilly topography environments, where pedestrian networks may not easily be established and added. In this longitudinal study we found that after the establishment of new pedestrian networks in a hilly environment, as measured by intersection density, people walked longer distance and their walking ratio also increased. These results provide evidence to suggest that even in hilly environments, walking can be promoted by increased pedestrian connectivity.

We found that changes to land use, including increasing the number work area buildings and life area buildings, influenced walking behaviors, but in different aspects. An increase in the number of work area buildings resulted in subjects having a decrease in the altitude ranges they walked, while the increased exposure to life area buildings encouraged subjects to take longer walking trips. For the destination-oriented walking findings in this study, newly established work area buildings mostly located in the lower height level of campus might serve as trip destinations thus decreasing walked altitude ranges. Increased life area buildings might provide more potential opportunities for walking trips. In addition, we noted that most of the newly constructed life area buildings were in locations closely connected to the newly established escalator. This may have a contributory effect on walking distance promotion. Our findings also highlight the need to parcel land use into distinct categories, as different land uses seem to influence different aspect of walking behaviors. However, very few studies have investigated the differential impact of different types of land uses [26-28]. Hirsch, J. A. and her colleagues used discrete land uses categorized by accessibility, intensity, and diversity to examine the influence on walking behaviors, and found small but non-significant associations in the expected direction between disaggregated land use measures and walking for transportation in New York. They postulate that individual components of land use patterns, which act as small components of the entire built environment, act in concert with other environmental features to encourage walking [28]. Our longitudinal study likewise provides new evidence that discrete land use categories may help identify unique influences on walking behavior.

We found that changes to “soft” infrastructure such as to campus bus service, significantly influenced walking behavior on campus. Notably, after changes in the bus schedules were implemented, students altered the altitude ranges they were willing to walk. We considered walking and bus riding behaviors together. We did not find statistically significant evidence for partition of the trips by travel mode. The results were in the expected direction, with an increase in regular bus schedules negatively influencing walking ratio, and a decrease in middle-class bus schedules positively influencing walking ratio. These findings might imply a competitive relationship between different travel modes (walking versus bus riding). Our findings highlight the importance of including both non-motorized (walking of cycling) and motorized (car driving or bus riding) transportation when assessing travel patterns, otherwise a potential exists to fail to capture the complete picture of walking behaviors. Recently, other work has also begun using both Walking and Transit scores to assess the impact of the built environment on walking patterns [29]. The categorization of bus services by intended function creates two different bus measures that avoid a zero-sum issue that could arise from a single bus measure which sums increases and decreases in services, potentially failing to capture changes to different types of services.

We found that an increase in population density was related to a significant increase in students’ walked altitude range, and overall walked distances. In the university’s hilly environment, it appears that population density played an important role on student’s walking choices. One possible explanation for our findings might be that as population density increases, crowding for bus transportation may intensify with a ceiling effect on ridership. In interviews we conducted with subject prior to this natural experiment, subjects reported a preference for walking if buses were crowded, even within the university’s hilly topography. Population density is a social aspect of the built environment, and decisions on whether to walk or ride a bus with friends may likewise be related to social environment influences [30].

The study has several limitations. Our study did not include a control group. Nevertheless, we found significant changes in walking behavior after changes to the built environment. We collected one follow-up survey in this study, and longer term effects on walking behavior are not clear [31]. Our walking diary was not validated, though it was adapted from established travel diaries [32] and was assessed for face validity with experts in urban planning and pilot tested on volunteers. We postulated a simplified assumption on the population density distribution which is an even distribution throughout the campus. The population density would likely change throughout the day due to student movement around campus. This study did not measure the exact locations of the entire student body at different times throughout the day. The study included students at a single university in Hong Kong and findings may not be generalizable. Subjects in our study were 18 to 20 years old students; their age and student status may influence their walking choices. Finally, though our built environment variables included distinct measureable changes in the physical infrastructure, it is still difficult to rule out the possibility that other coinciding changes in the built environment may not also have played a role in the observed changes in walking behavior. This possibility of residual confounding makes it somewhat difficult to give clear guidance to urban planners or public health practitioners.

Despite these limitations, our study has important strengths. The longitudinal survey design provides insight into causal relationships. To our knowledge, there are few longitudinal studies that combine before-and after survey data with measured changes to the built environment, to study changes in walking behavior. Krizek K. J. used longitudinal household travel data in Seattle to examine the relationship between changes in neighborhood and household travel behavior. The results show that after controlling for changes in lifestyle, relocating households to neighborhoods with more accessibility could effectively reduce vehicle miles traveled, but found no significant effect on trip generation or travel mode [9]. Cao and colleagues studied people who relocated in Northern California and identified promising neighborhood characteristic after controlling for “self-selection” (individuals who like to walk move to more walkable areas), however, recall bias after residential relocation remained possible [10]. Natural experiments, especially those involving changes to the built environment are rare and difficult to anticipate, which can also make it difficult to follow a cohort of subjects over time. Our longitudinal study with a built environment natural experiment provides empirical evidence on how changes to the built environment are associated with changes in walking behavior.

## Conclusions

In summary, we leveraged a natural experiment to study changes in the built environment and test how these changes were associated with changes in walking behavior among a cohort of subjects exposed to the built environment changes. The individual-based measurements of exposure to the built environment we developed in this study are a feasible and effective way to contextualize and test the interaction between subjects and their walking environments. Changes to the built environment at a university campus in Hong Kong had important and measurable effects on walking behaviors, including distances walked, proportion of trips walked (versus vehicular trips), and altitude ranges walked. The use of a natural experiment coupled with pre-and-post longitudinal data collection methods provides some insight into the causal relationships between the built environment and walking behaviors and has implications for planning, health, and public policy.

## Competing interests

The authors declare they have no competing interests.

## Authors’ contributions

GS drove the conceptualization of the study design, undertook the analysis, and wrote the first draft of this paper under the guidance of NMO. NMO made important contributions to the study design, statistical analysis and redrafting of the paper. HL contributed to the study design and the Geospatial analysis. All authors read and approved the final manuscript.
